# Cardiac remodeling and myocardial late gadolinium enhancement in competitive amateur cyclists: insights from cardiac MRI

**DOI:** 10.1007/s10554-026-03693-x

**Published:** 2026-04-13

**Authors:** Felix Albrecht, Tilo Thottakara, Jonas Schaerk, Goetz H. Welsch, Julia Münch, Axel Pressler, Scott B. Reeder, Monica Patten, Gunnar K. Lund, Jitka Starekova

**Affiliations:** 1 Department of Diagnostic and Interventional Radiology and Nuclear Medicine, University Medical Center Hamburg-Eppendorf, Hamburg, Germany; 2 Department of Trauma Surgery, Orthopedics and Sports Traumatology, BG Klinikum Hamburg, Hamburg, Germany; 3 Department of Cardiology, University Heart and Vascular Center Hamburg, University Medical Center Hamburg-Eppendorf, Hamburg, Germany; 4 Department of Cardiology, University of California, San Francisco, San Francisco, USA; 5 Athleticum, University Medical Center Hamburg-Eppendorf, Hamburg, Germany; 6 Department of Trauma and Orthopaedic Surgery, University Medical Center Hamburg- Eppendorf, Hamburg, Germany; 7 Private Center for Sports and Exercise Cardiology, Munich, Germany; 8 Department of Medical Physics, Biomedical Engineering, Medicine and Emergency Medicine, University of Wisconsin-Madison, Madison, USA; 9 Department of Radiology, University of Wisconsin-Madison, 1111 Highland Ave, WI 53705 Madison, USA

**Keywords:** Myocardial fibrosis, Competitive cycling, Cardiac magnetic resonance, Athlete’s heart, Late gadolinium enhancement, RVIP LGE

## Abstract

**Purpose:**

To evaluate cardiac remodeling and right ventricular insertion point (RVIP) and non-RVIP late gadolinium enhancement (LGE) patterns, and their clinical and performance correlates, in cyclists using MRI.

**Methods:**

In this prospective study, 66 competitive amateur cyclists (88% male; age 42 ± 13y) and 35 controls (74% males; age 40 ± 13y) underwent 3.0T contrast-enhanced cardiac MRI and cardiopulmonary exercise testing. Cycling history and competitiveness (score:10–70; higher=greater drive) were assessed by questionnaire.

**Results:**

Compared to controls, cyclists showed ‘athlete’s heart’ characteristics, including bi-ventricular enlargement and increased left ventricular (LV) mass (all *p* < 0.001). Focal non-ischemic myocardial LGE occurred in 19/58 (33%) of male cyclists, predominantly at the RVIP (RVIP LGE, 79%), and inferolateral wall (non-RVIP LGE, 21%). No female cyclists showed LGE. Male cyclists with non-RVIP LGE had higher myocardial mass (90 ± 5 vs. 79 ± 8 g/m^2^, *p* = 0.013), reduced RV ejection fraction (RVEF, 47 ± 4% vs. 55 ± 5%, *p* = 0.031), and elevated high-sensitivity troponin T (8.8 ± 1.7 vs. 5.1 ± 2.0 pg/ml, *p* = 0.016) compared to those without LGE. Compared to RVIP LGE cyclists, they also had greater LV mass (90 ± 5 vs. 74 ± 10 g/m^2^, *p* = 0.001), higher LV end-diastolic volume (133 ± 16 vs. 108 ± 12 ml/m^2^, *p* = 0.043) and lower RVEF (47 ± 4% vs. 57 ± 5%, *p* = 0.011). Cyclists with RVIP LGE had comparable myocardial mass as those without LGE (*p* = 0.082). LGE presence was not associated with maximal oxygen uptake, exercise systolic blood pressure, cycling history, or competitiveness (all *p* > 0.05).

**Conclusion:**

Nearly one-third of cyclists had focal non-ischemic myocardial LGE, predominantly at the RVIP. Adverse remodeling and reduced function were confined to cyclists with non-RVIP LGE, suggesting a distinct, pathological phenotype.

**Supplementary Information:**

The online version contains supplementary material available at 10.1007/s10554-026-03693-x.

## Introduction

Cardiac adaptation and potential negative impacts of high-volume endurance training on athletes’ hearts remain a topic of ongoing research [1, 2]. Regular endurance exercise induces a benign cardiac remodeling phenotype, characterized by increases in chamber size and myocardial mass with preserved function, commonly referred to as ‘athlete’s heart’ [3]. While exercise offers significant cardiovascular benefits [4], excessive training has been linked to permanent cardiac damage and myocardial fibrosis, which can serve as a substrate for arrhythmias [1, 5, 6]. It has also been associated with the rare occurrence of sudden cardiac death [7, 8].

Myocardial fibrosis can be detected using contrast-enhanced cardiac magnetic resonance imaging (MRI) [5]. Focal fibrosis appears as areas of late gadolinium enhancement (LGE) in the myocardium, while diffuse myocardial fibrosis is evaluated with T1 mapping, showing increased native T1 relaxation times and elevated extracellular volume (ECV) values [9–11].

The prevalence of focal non-ischemic myocardial fibrosis detected by cardiac MRI in otherwise healthy, asymptomatic endurance athletes ranges from 0 to 50%, depending on athletic population and type of sport [5, 12]. Though the underlying mechanisms are not well understood, some evidence suggests a link to excessive training [6], and undiagnosed myocarditis has also been postulated as a contributing factor [13]. Strenuous, prolonged exercise and athletes’ drive to compete may lead them to train through illness, increasing the risk of myocarditis [14].

Most cardiac MRI research in endurance athletes has focused on triathletes and runners, with cyclists often underrepresented or included in mixed-athletes cohorts only [1, 2, 15]. However, cycling imposes distinct hemodynamic demands compared to running or swimming [16]. Intense lower body muscle engagement and sustained contractions during seated cycling, can increase peripheral vascular resistance, leading to elevated systemic blood pressure during exercise. Notably, an exaggerated peak systolic blood pressure response to cycle ergometry has been linked to future hypertension in normotensive individuals [17–19].

In triathletes, total cycling distance during competitions and peak systolic blood pressure were independent predictors of focal myocardial fibrosis, suggesting a potential sport-specific contribution of cycling to myocardial injury [6]. Furthermore, an exercise-induced increase in T1 relaxation time following a cycling event further supports this concept [20]. Despite these observations, cycling-specific cardiac MRI studies remain limited, often including only males, and frequently lacking detailed training histories [15, 20, 21].

Two distinct patterns of non-ischemic LGE have been described in endurance athletes: fibrosis at the right ventricular insertion points (RVIP) and non-RVIP fibrosis, often subepicardial or mid-myocardial, primarily affecting the inferolateral left ventricular (LV) wall. While historically grouped together, recent mixed-athlete studies suggest these patterns may have divergent clinical implications, particularly regarding arrhythmogenic risk [1, 2]. Whether these LGE patterns reflect distinct structural phenotypes or biomarkers of subclinical injury in competitive cyclists remains unknown.

Therefore, the purpose of this study was to assess cardiac adaptations and LGE patterns in competitive amateur cyclists using cardiac MRI. We aimed to determine whether distinct LGE patterns are associated with divergent structural, functional and biochemical profiles, and whether training load and competitiveness influence their presence and distribution.

## Materials and methods

### Participants

This prospective study, approved by the institutional ethics committee in accordance with the Declaration of Helsinki (Protocol code PV4764-3108-BO-ff, date of approval July 29, 2014), recruited competitive amateur cyclists and healthy controls between March 1, 2018, and October 31, 2019. Informed consent was obtained. Cyclists were identified through advertisements at local, registered cycling clubs.

Participants were included if they met the following criteria, based on the standardized self-reported questionnaire: (a) regularly participation in cycling competitions across various distances, and (b) cycling at least 9,000 km in the past year and/or training on average of at minimum 10 h per week. Study exclusion criteria included contraindications for cardiac MRI, any cardiovascular or systemic diseases, and use of illicit drugs.

Cyclists were asked to report their lifetime competition history, years of competitive cycling, weekly exercise volume (in hours), and number of cycling competitions per year using a standardized questionnaire. Cyclists’ intrinsic motivation to compete was assessed using a study-specific questionnaire (Supplementary Material, Table S1) that evaluated individual’s motivation to compete and their competitive drive (minimum-maximum points: 10–70; higher score- more win-oriented, strong competitive drive).

Control participants were included if healthy and if they averaged less than 3 h of exercise per week. Controls were subject to the same exclusion criteria as the cyclists, and ineligible if they engaged in regular structured athletic training.

Prior to cardiac MRI exams, all subjects were instructed to refrain from exercise for 72 h. Prior to cardiac MRI blood samples were drawn from an antecubital vein to obtain hematocrit and markers of cardiac injury, including high-sensitivity troponin T (hs-TnT), creatine kinase (CK) and N-terminal pro–B-type natriuretic peptide (NT-proBNP). Body surface area (BSA) and body mass index (BMI) were calculated from the study participant’s body weight and height.

We note that this cyclist collective was included in a previous report focusing on prognostic outcomes in a mixed-athlete population [8]. The current manuscript represents the primary, detailed investigation of this specific cohort, providing cyclist-specific data and analysis not addressed in the prior aggregate study.

### Cardiac MRI Protocol

Cardiac MRI exams were performed using a 3.0T clinical MRI system (Achieva, Philips Healthcare, Netherlands) with 32-channel cardiac phased array receiver coil. The protocol (Supplementary Material, Table S2) included gated CINE imaging using balanced steady-state free-precession (bSSFP) in the short axis for measuring LV and right ventricle (RV) volumes and LV mass.

T1 mapping was performed using a Modified Look Locker Inversion Recovery (MOLLI) sequence on 3 double-oblique left ventricular short-axes (SA) slices (apical, mid, and basal) before and 15 min after administration of 0.2mmol/kg gadoterate meglumine (Dotarem, Guerbet, Sulzbach, Germany; rate 2.5 ml/s). T2 mapping was performed using a black-blood, multi-echo GRASE-based sequence on 3 double-oblique left ventricular SA slices (apical, mid, and basal) prior to contrast administration. T1- and T2 maps were generated using a third-party cardiovascular software (cvi42, Circle Cardiovascular Imaging Inc., Calgary, Alberta, Canada).

For LGE imaging, end-diastolic images were acquired 10 min after contrast administration using phase-sensitive inversion recovery (PSIR) sequence in SA orientation covering the entire heart and in standard long-axis views (2-,3- and 4-chamber).

### Cardiac MRI Analysis

Two observers, a physician (FA) under the supervision of radiologist with 13 years cardiac MRI experience (JS), and a cardiologist with 7 years cardiac MRI experience (TT) both blinded to clinical data, performed image analysis using cvi42 software (Circle Cardiovascular Imaging Inc, Calgary, Alberta, Canada). For volumetric and mass analysis, segmentation of the LV and RV endocardial and epicardial borders was performed using semi-automated contouring, with manual adjustments in standard fashion on SA images [22]. As part of the segmentation, papillary muscles and trabeculae were excluded from the LV mass but included in the LV volume in the primary analysis. All measurements were indexed to BSA. A secondary analysis was performed by a single experienced radiologist (JS) using semi-automated contouring with manual adjustments to reclassify papillary muscles and prominent trabeculations as part of the myocardial mass to allow comparison with prior athletic cohort studies using this type of segmentation.

Native T1, T2, and post-contrast T1 times were measured using a single mid-septal region of interest (ROI) on midventricular short-axis images, excluding areas of focal LGE if present. ECV was calculated using the standard formula [9]. Results are given as the mean of two observers’ measurements.

Focal myocardial fibrosis was defined as an area of LGE present in at least 3 consecutive SA images, or confirmed on perpendicular long-axis images, ensuring the findings represents a significant tissue volume rather than minor transient artifacts at the RVIP of LV or elsewhere. To maximize diagnostic accuracy and eliminate inter-observer variability all images were reviewed in a side-by-side consensus agreement by two observers with 13- and 20-years cardiac MRI experience (JS and GKL), respectively. Athletes with LGE present exclusively at the RV insertion point (RVIP) were classified as RVIP LGE cyclists. Those with LGE in other areas, or in both the RVIP and other locations, were classified as non-RVIP LGE cyclists. Accordingly, non-RVIP LGE was considered the primary phenotype of interest for pathological remodeling analyses, whereas RVIP LGE was analyzed descriptively as a comparator pattern. Quantitative analysis of LGE was performed on SA LGE images by one observer (JS) using a threshold method, with a cutoff of more than 5 standard deviations (SD) above remote, healthy myocardium.

### Cardiopulmonary Exercise Test

A cardiopulmonary exercise test was performed using a ramp-incremental protocol on an eddy current-braked, high-performance, sport-specific cycle ergometer (Lode Excalibur Sport 911900, Lode BV, Groningen, Netherlands). Subjects wore an appropriately sized silicone oronasal face mask that was securely fitted to their heads using polyurethane headgear (7450 SeriesV2 MaskTM, Hans Rudolph, Inc., Kansas, USA).

The ramp-incremental protocol differed for males and females to ensure all subjects reached maximum exhaustion within 10–12 min. The starting load was 50 W, with a continuous increase of 40 W/min for males and 30 W/min for females. Total exhaustion was defined as a cycling cadence falling below 50 rpm. Breath-by-breath gas exchange analysis was performed throughout the exercise test, to determine maximal oxygen uptake (VO_2_max), a measure of the participant’s cardiorespiratory capacity and endurance, along with the maximal power output (W) at exhaustion [23].

A 12-lead electrocardiogram (Custo Cardio 100 and 300, Custo Med GmbH, Ottobrunn, Germany) was continuously recorded at rest, during the ramp-incremental protocol, and for 5 min post-exercise. Blood pressure was measured manually every two minutes. Resting and peak systolic blood pressure during exercise, along with heart rate, were recorded.

In addition, body fat was measured using a Harpenden Skinfold Caliper (Baty International, Burgess Hill, England). Skinfold thickness at the ten sites required for the Parizkova method was measured to the nearest 0.1 mm, and body fat was estimated using sex-specific Parizkova’s Eqs [24, 25].

### Statistical Analysis

Statistical analysis was performed using the packages table1 (v1.4.3) and tidyverse (v2.0.0) in R (version 4.1.1). Continuous data are presented as mean ± SD, and categorical data are presented as absolute numbers and percentages. Continuous data were compared using two-sided Student’s t tests, and categorical variables were compared using chi-square or Fisher exact test, as appropriate. Given the exploratory nature of the subgroup analyses and the limited sample sizes in specific cohorts, p-values were not adjusted for multiple comparisons to minimize the risk of Type II errors (false negatives) and avoid masking potentially relevant physiological signals [26]. However, Cohen’s d effect sizes are provided to contextualize the magnitude of observed differences.

**Table 1:** Characteristics of male and female cyclists and controls

-	Male controls (*n* = 26)	Male cyclists (*n* = 58)	*P*-value	Female controls (*n* = 9)	Female cyclists (*n* = 8)	*P*-value
Clinical parameters						
Age, yrs	41 ± 12	43 ± 12	0.457	38 ± 14	35 ± 13	0.742
Weight, kg	84 ± 12	79 ± 9	0.0653	62 ± 9	65 ± 10	0.466
Height, m	1.81 ± 0.08	1.81 ± 0.06	0.706	1.67 ± 0.05	1.74 ± 0.07	0.063
BMI, kg/m²	25.6 ± 3.4	23.9 ± 2.6	0.0264	22.0 ± 3.3	21.4 ± 2.1	0.659
BSA, m²	2.04 ± 0.16	1.99 ± 0.13	0.197	1.69 ± 0.1	1.78 ± 0.17	0.221
Body fat, %	19 ± 4	16 ± 5	0.003	20 ± 3	17 ± 4	0.094
Hematocrit, %	43 ± 3	44 ± 3	0.414	37 ± 4	40 ± 2	0.130
hs-TnT, pg/ml	5.6 ± 2.1	5.8 ± 2.2	0.777	5.8 ± 3.0	4.6 ± 1.1	0.434
NT-proBNP, pg/ml	30.2 ± 26.4	30.1 ± 32.6	0.998	50.1 ± 39.1	40.6 ± 17.8	0.525
CK, U/l	196 ± 173	164 ± 166	0.437	82 ± 36	121 ± 85	0.261
Exercise test						
Systolic BP at rest, mmHg	124 ± 9	124 ± 10	0.963	113 ± 13	117 ± 9	0.434
Diastolic BP at rest, mmHg	80 ± 2	80 ± 5	0.622	76 ± 7	81 ± 3	0.084
Peak systolic BP, mmHg	181 ± 23	211 ± 24	< 0.001	158 ± 13	187 ± 24	0.013
Peak diastolic BP, mmHg	80 ± 2	84 ± 7	< 0.001	78 ± 7	87 ± 18	0.228
Heart rate at rest, bpm	61 ± 11	52 ± 8	< 0.001	61 ± 7	55 ± 7	0.125
Peak heart rate, bpm	176 ± 13	180 ± 14	0.24	177 ± 9	182 ± 15	0.458
VO_2_max , ml/kg/min	41 ± 8	57 ± 10	< 0.001	35 ± 8	52 ± 11	0.004
Maximal power, W	346 ± 58	430 ± 53	< 0.001	218 ± 60	317 ± 31	0.002
Cardiac MRI parameters						
LVEF, %	60 ± 3	61 ± 5	0.17	64 ± 4	64 ± 6	0.979
LV mass index, g/m²	59 ± 9	79 ± 9	< 0.001	48 ± 6	61 ± 7	< 0.001
LVEDVi, ml/m²	96 ± 17	113 ± 12	< 0.001	79 ± 9	100 ± 9	< 0.001
LVESVi, ml/m²	39 ± 8	44 ± 8	0.0079	29 ± 4	36 ± 8	0.031
LVSVi, ml/m²	57 ± 9	69 ± 8	< 0.001	50 ± 6	64 ± 7	< 0.001
LAVi, ml/m²	34 ± 9	40 ± 11	0.006	29 ± 4	37 ± 9	0.034
RVEF, %	57 ± 4	55 ± 5	0.124	58 ± 5	56 ± 6	0.394
RVEDVi, ml/m²	101 ± 17	125 ± 17	< 0.001	82 ± 11	107 ± 9	< 0.001
RVESVi, ml/m²	44 ± 9	57 ± 13	< 0.001	35 ± 8	47 ± 9	0.008
RVSVi, ml/m²	57 ± 10	68 ± 8	< 0.001	48 ± 6	60 ± 6	0.002
RAVi, ml/m²	38 ± 10	52 ± 16	< 0.001	35 ± 9	46 ± 13	0.058
LGE present, n (%)	4 (15.4%)	19 (32.8%)	0.119	0 (%)	0 (%)	-
LGE size, %LV	3.0 ± 3.3	2.0 ± 2.6	0.6	-	-	-
LGE mass index, g/m²	1.5 ± 1.6	1.3 ± 2.0	0.858	-	-	-
Native T1, ms	1230 ± 39	1220 ± 37	0.308	1260 ± 33	1170 ± 130	0.109
Native T2, ms	46 ± 4	47 ± 4	0.302	44 ± 3	45 ± 4	0.475
ECV, %	26 ± 4	26 ± 4	0.605	31 ± 4	27 ± 3	0.064

Numbers are mean ± SD for continuous and n (%) for categorical data. Notes: BMI, body mass index; BSA, body surface area; BP, blood pressure; CK, Creatine kinase; ECV, extracellular volume; HR, heart rate, hs-TnT, high-sensitive troponin T; LAVi, Left atrial end-systolic volume index, LV, left ventricular; LGE, late gadolinium enhancement; LVEF, left ventricular ejection fraction; LVEDVi, left ventricular end-diastolic volume index; LVESVi, left ventricular end-systolic volume index; LVSVi, left ventricular stroke volume index; RAVi, Right atrial end-systolic volume index; RVEF, right ventricular ejection fraction; RVEDVi, right ventricular end-diastolic volume index; RVESVi, right ventricular end-systolic volume index; RVSVi, right ventricular stroke volume index; NT-proBNP, N-terminal pro-brain natriuretic peptide; VO_2_ max, maximal oxygen uptake

## Results

**Fig. 1:**
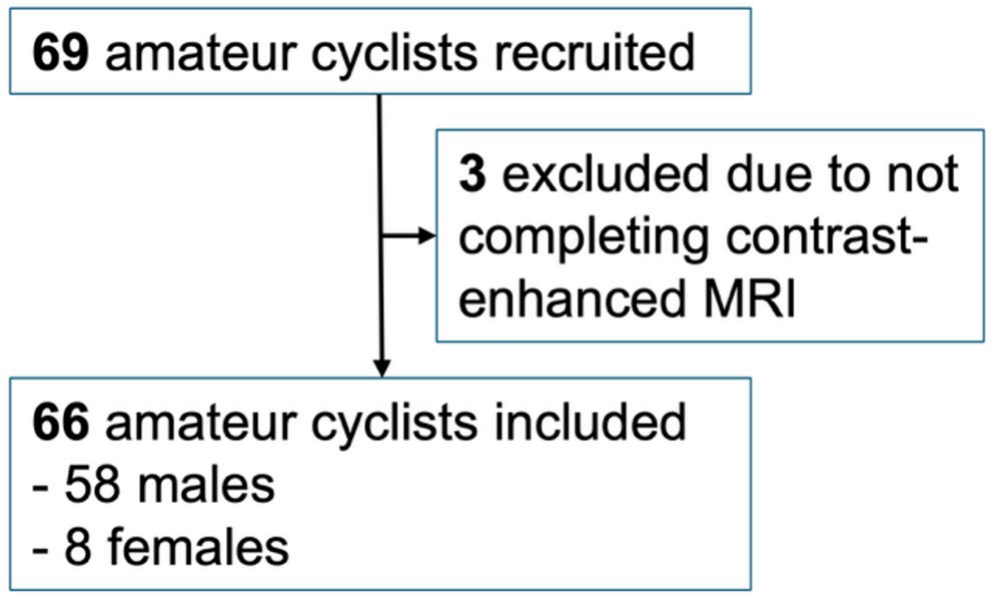
Flow diagram of cyclists’ cohort. A total of 69 competitive amateur cyclists were recruited. Three cyclists were excluded from the analysis because they did not complete the contrast-enhanced MRI due to claustrophobia and contrast allergy. The final study population included 66 competitive amateur cyclists, 58 males and 8 females

A total of 69 competitive amateur cyclists were recruited for this study. Three cyclists were excluded from the analysis because they did not complete the contrast-enhanced MRI due to claustrophobia (*n* = 2) or contrast allergy (*n* = 1) (Fig. 1). The final study population included 66 competitive amateur cyclists (58 males, mean age 43 ± 12 years; 8 females, mean age 35 ± 13 years) and 35 control participants (26 males, mean age 41 ± 12 years; 9 females, mean age 38 ± 14 years) (Table 1).

### Cyclists vs. controls

Male cyclists and controls had similar age and BSA (Table 1). Male controls had a higher BMI (25.6 ± 3.4 kg/m² vs. 23.9 ± 2.6 kg/m², *p* = 0.026) and body fat percentage (19 ± 4% vs. 16 ± 5%, *p* = 0.003) compared to cyclists. Age, BSA, BMI and body fat percentage were similar for female cyclists and controls (Table 1). Cardiac markers and hematocrit did not differ between the sex-matched groups (Table 1). No relevant ECG abnormalities were observed.

Cyclists, both males and females, showed superior maximal power outputs compared to the sex-matched controls (males: 430 ± 53 W vs. 346 ± 58 W, *p* < 0.001; females: 317 ± 31 W vs. 218 ± 60 W, *p* = 0.002). VO_2_max was higher in cyclists than in controls (males: 57 ± 10 ml/kg/min vs. 41 ± 8 ml/kg/min, *p* < 0.001; females: 52 ± 11 ml/kg/min vs. 35 ± 8 ml/kg/min, *p* = 0.004). Cyclists had higher peak systolic blood pressure compared to controls (males: 211 ± 24mmHg vs. 181 ± 23mmHg, *p* < 0.001; females: 187 ± 24 vs. 158 ± 13mmHg, *p* = 0.013). Blood pressure at rest did not differ between the groups (Table 1).

Cyclists showed increased ventricular dimensions (Table 1). LV end-diastolic volume (LVEDVi) was greater in both male and female cyclists compared to sex-matched controls (males: 113 ± 12 ml/m² vs. 96 ± 17 ml/m², *p* < 0.001; females: 100 ± 9 ml/m² vs. 79 ± 9 ml/m², *p* < 0.001). Similarly, RV end-diastolic volume (RVEDVi) was greater in cyclists compared to sex-matched controls (males: 125 ± 17 ml/m² vs. 101 ± 17 ml/m², *p* < 0.001; females: 107 ± 9 ml/m² vs. 82 ± 11 ml/m², *p* < 0.001). Cyclists had higher LV mass compared to controls (males: 79 ± 9 g/m² vs. 59 ± 9 g/m², *p* < 0.001; females: 61 ± 7 g/m² vs. 48 ± 6 g/m², *p* < 0.001). The LV- and RV ejection fraction were similar between the groups.

Myocardial LGE was present in 4 of 26 (15.4%) male controls (Table 1). In all 4 cases, the LGE was small and nonspecific, limited to the LV septum at the inferior RVIP. One of the controls also had midmyocardial LGE in the basal inferolateral LV wall.

**Fig. 2:**
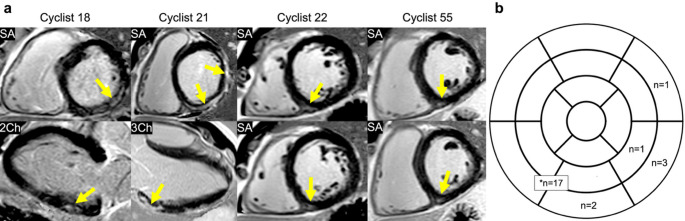
Representative late gadolinium enhancement (LGE) patterns and schematic distribution in male cyclists. (a) Representative short-axis images from four male cyclists showing nonspecific LGE of the left ventricle (LV) at the inferior right ventricular insertion point (RVIP; Cyclists 22 and 55) and non-ischemic midmyocardial or subepicardial LGE (Cyclists 18 and 21). (b) Schematic representation of segmental LGE distribution in male cyclists. The most common site (*n* = 17, marked by asterisk) was the RVIP (basal and/or midventricular). In addition, four cyclists showed non-ischemic midmyocardial or epicardial LGE involving one or more segments, predominantly in the basal inferolateral left ventricular wall. No female cyclists exhibited myocardial LGE

Among male cyclists, 19 of 58 male cyclists (32.8%) showed LGE (Table 1). Of these 15 had small, nonspecific LGE limited to the LV septum at the inferior RVIP. The remaining four cyclists had non-ischemic midmyocardial LGE mainly in the basal inferolateral LV wall (Fig. 2); 2 of these, also had LGE at the inferior RVIP. No female cyclists or controls showed myocardial LGE.

Native T1 time, ECV and T2 values did not differ between cyclists and the controls (Table 1).

### Male cyclists with LGE vs. without LGE

The mean age, BSA, BMI and body fat were similar between cyclists with and without LGE (Table 2). Hs-TnT levels were higher in cyclists with LGE compared to those without LGE (7.0 ± 2.0pg/ml vs. 5.1 ± 2.0pg/ml, *p* = 0.004), while NT-proBNP and CK levels showed no significant differences (Table 2). Exercise test parameters, including VO_2_max, maximal power output, and peak systolic blood pressure during exercise were similar between the groups (Table 2). Volumetric and mapping parameters did not differ significantly (Table 2). There were no differences in the cycling history or competitiveness scores between cyclists with and without LGE (Table 2).

**Table 2:** Comparison of male cyclists with (LGE+) and without LGE (LGE-)

-	LGE – cyclists (*n* = 39)	LGE + cyclists (*n* = 19)	*P*-value
Clinical parameters			
Age, yrs	43 ± 14	43 ± 7	0.902
Weight, kg	78 ± 10	80 ± 9	0.37
Height, m	1.81 ± 0.06	1.82 ± 0.06	0.525
BMI, kg/m²	23.7 ± 2.6	24.2 ± 2.6	0.545
BSA, m²	1.98 ± 0.13	2.01 ± 0.12	0.336
Body Fat, %	16 ± 5	15 ± 6	0.727
Hematocrit, %	44 ± 3	43 ± 3	0.51
hs-TnT, pg/ml	5.1 ± 2.0	7.0 ± 2.0	0.004
NT-proBNP, pg/ml	28.2 ± 18.1	34.2 ± 51.9	0.643
CK, U/l	165 ± 197	163 ± 75	0.953
Exercise test			
Systolic BP at rest, mmHg	125 ± 10	121 ± 8	0.169
Diastolic BP at rest, mmHg	79 ± 4	81 ± 6	0.245
Peak systolic BP, mmHg	213 ± 26	207 ± 18	0.257
Peak diastolic BP, mmHg	83 ± 6	85 ± 8	0.285
Heart rate at rest, bpm	53 ± 8	50 ± 8	0.137
Peak heart rate, bpm	180 ± 14	180 ± 15	0.917
VO_2_max, ml/kg/ min	57 ± 10	57 ± 10	0.98
Maximal power, W	427 ± 55	435 ± 49	0.583
Cardiac MRI parameters			
LVEF, %	61 ± 4	61 ± 6	0.652
LV mass index, g/m^2^	79 ± 8	78 ± 11	0.511
LVEDVi, ml/m²	112 ± 9	113 ± 16	0.81
LVESVi, ml/m²	43 ± 6	45 ± 11	0.615
LVSVi, ml/m²	69 ± 7	69 ± 11	0.892
LAVi, ml/m²	40 ± 11	41 ± 12	0.685
RVEF, %	55 ± 5	55 ± 6	0.9
RVEDVi, ml/m²	125 ± 13	125 ± 22	0.978
RVESVi, ml/m²	56 ± 10	57 ± 17	0.867
RVSVi, ml/m²	68 ± 7	68 ± 9	0.816
RAVi, ml/m²	53 ± 14	49 ± 20	0.369
Native T1, ms	1210 ± 32	1230 ± 43	0.149
Native T2, ms	47 ± 3	47 ± 5	0.359
ECV, %	25 ± 3	26 ± 6	0.618
Cycling history			
Active years, n	14 ± 10	15 ± 11	0.705
Exercise volume, hours/week	10 ± 3	11 ± 5	0.429
Competitions n/year	15 ± 14	12 ± 9	0.217
Mean km/year	9370 ± 2880	9300 ± 4590	0.953
Competitiveness score	58 ± 14	57 ± 13	0.887

Numbers are mean ± SD for continuous and n (%) for categorical data. Notes: BMI, body mass index; BSA, body surface area; BP, blood pressure; CK, Creatine kinase; ECV, extracellular volume; HR, heart rate, hs-TnT, high-sensitive troponin T; LAVi, Left atrial end-systolic volume index; LV, left ventricular; LGE, late gadolinium enhancement; LVEF, left ventricular ejection fraction; LVEDVi, left ventricular end-diastolic volume index; LVESVi, left ventricular end-systolic volume index; LVSVi, left ventricular stroke volume index; RAVi, Right atrial end-systolic volume index; RVEF, right ventricular ejection fraction; RVEDVi, right ventricular end-diastolic volume index; RVESVi, right ventricular end-systolic volume index; RVSVi, right ventricular stroke volume index; NT-proBNP, N-terminal pro-brain natriuretic pepetide; VO_2_max, maximal oxygen uptake

Cyclists with non-RVIP LGE (*n* = 4) had higher myocardial mass (90 ± 5 g/m^2^ vs. 79 ± 8 g/m^2^, *p* = 0.013) and lower RV EF (47 ± 4% vs. 55 ± 5%, *p* = 0.031) compared to cyclists without LGE (*n* = 39). They also showed a trend toward increased LV end-diastolic volume (133 ± 16 ml/m² vs. 112 ± 9 ml/m², *p* = 0.077), end-systolic volume (56 ± 9 ml/m², vs. 43 ± 6 ml/m², *p* = 0.065), and left atrium volume (48 ± 7 ml/m², vs. 40 ± 11 ml/m², *p* = 0.072) (Table 3). Additionally, non-RVIP LGE cyclists had higher hs-TnT (8.8 ± 1.7pg/ml vs. 5.1 ± 2.0pg/ml, *p* = 0.016), while NT-proBNP and CK levels were similar (Table 3). Exercise test parameters, cycling history, and competitiveness scores were comparable between the two groups (Table 3). Supplementary material, Table S3 provides LV mass and remodeling parameters with papillary muscles included in the myocardial mass.

**Table 3:** Comparison of male cyclists with non-RVIP LGE and without LGE (LGE-)

-	Non-RVIP LGE cyclists (*n* = 4)	LGE – cyclists (*n* = 39)	*P*-value	Cohen’s d
Clinical parameters				
Age, yrs	47 ± 9	43 ± 14	0.448	0.31
Weight, kg	84 ± 14	78 ± 10	0.427	0.64
Height, m	1.79 ± 0.02	1.81 ± 0.06	0.273	0.30
BMI, kg/m²	26.2 ± 4.2	23.7 ± 2.6	0.328	0.89
BSA, m²	2.03 ± 0.14	1.98 ± 0.13	0.526	0.39
Body Fat, %	17 ± 9	16 ± 5	0.877	0.14
Hematocrit, %	43 ± 3	44 ± 3	0.69	0.26
hs-TnT, pg/ml	8.8 ± 1.7	5.1 ± 2.0	0.016	1.80
NT-proBNP, pg/ml	36.8 ± 49.7	28.2 ± 18.1	0.755	0.39
CK, U/l	182 ± 50	165 ± 197	0.681	0.09
Exercise test				
Systolic BP at rest, mmHg	118 ± 10	125 ± 10	0.223	0.74
Diastolic BP at rest, mmHg	80 ± 8	79 ± 4	0.88	0.15
Peak systolic BP, mmHg	213 ± 15	213 ± 26	0.92	0.04
Peak diastolic BP, mmHg	83 ± 5	83 ± 6	0.899	0.06
Heart rate at rest, bpm	47 ± 12	53 ± 8	0.39	0.72
Peak heart rate, bpm	168 ± 25	180 ± 14	0.419	0.80
VO_2_max, ml/kg/min	57 ± 2	57 ± 10	0.865	0.04
Maximal power, W	429 ± 50	427 ± 55	0.954	0.03
Cardiac MRI parameters				
LVEF, %	57 ± 7	61 ± 4	0.354	0.88
LV mass index, g/m^2^	90 ± 5	79 ± 8	0.013	1.39
LVEDVi, ml/m²	133 ± 16	112 ± 9	0.077	2.17
LVESVi, ml/m²	56 ± 9	43 ± 6	0.065	1.96
LVSVi, ml/m²	77 ± 16	69 ± 7	0.416	0.98
LAVi, ml/m²	48 ± 7	40 ± 11	0.072	0.84
RVEF, %	47 ± 4	55 ± 5	0.031	1.60
RVEDVi, ml/m²	144 ± 36	125 ± 13	0.377	1.17
RVESVi, ml/m²	76 ± 26	56 ± 10	0.228	1.65
RVSVi, ml/m²	67 ± 12	68 ± 7	0.896	0.11
RAVi, ml/m²	59 ± 40	53 ± 14	0.796	0.33
Native T1, ms	1240 ± 68	1210 ± 32	0.447	0.82
Native T2, ms	47 ± 1	47 ± 3	0.875	0.04
ECV, %	28 ± 1	25 ± 3	0.717	0.51
Cycling history				
Active years, n	23 ± 21	14 ± 10	0.428	0.87
Exercise Volume, h per week	11 ± 6	10 ± 3	0.614	0.52
Competitions per year, n	17 ± 16	15 ± 14	0.847	0.12
Mean km/year	14200 ± 6800	9370 ± 2880	0.251	1.45
Competitiveness score	55 ± 17	58 ± 14	0.769	0.19

Numbers are mean ± SD for continuous and n (%) for categorical data. Cohen’s d indicates effect size (0.2 = small, 0.5 = medium, 0.8 = large, very large > 1.2) Notes: BMI, body mass index; BSA, body surface area; BP, blood pressure; CK, Creatine kinase; ECV, extracellular volume; HR, heart rate, hs-TnT, high-sensitive troponin T; LAVi, Left atrial end-systolic volume index; LV, left ventricular; LGE, late gadolinium enhancement; LVEF, left ventricular ejection fraction; LVEDVi, left ventricular end-diastolic volume index; LVESVi, left ventricular end-systolic volume index; LVSVi, left ventricular stroke volume index; RAVi, Right atrial end-systolic volume index; RVEF, right ventricular ejection fraction; RVEDVi, right ventricular end-diastolic volume index; RVESVi, right ventricular end-systolic volume index; RVSVi, right ventricular stroke volume index; NT-proBNP, N-terminal pro-brain natriuretic pepetide; VO_2_max, maximal oxygen uptake

Cyclists with RVIP LGE (*n* = 15) had similar MRI parameters, compared to those without LGE (Supplementary Material, Table S4). Exercise test results and cycling history were also comparable between the groups. However, RVIP LGE cyclists showed slightly higher hs-TnT levels (6.5 ± 1.8 pg/ml vs. 5.1 ± 2.0 pg/ml, *p* = 0.040), while NT-proBNP and CK levels were similar (Supplementary Material, Table S4).

### Male cyclists with RVIP LGE vs. non-RVIP LGE

The mean age, BSA, and BMI were not significantly different between cyclists with RVIP LGE and those with non-RVIP LGE.

Cyclists with non-RVIP LGE (*n* = 4) had higher myocardial mass (90 ± 5 g/m^2^ vs. 74 ± 10 g/m^2^, *p* = 0.001), lower right ventricular ejection fraction (RVEF) (47 ± 4% vs. 57 ± 5%, *p* = 0.011), increased LV end-diastolic volume (133 ± 16 ml/m² vs. 108 ± 12 ml/m², *p* = 0.043), and end-systolic volume (56 ± 9 ml/m² vs. 42 ± 9 ml/m², *p* = 0.040), and a trend toward increased left atrium volume (48 ± 7 ml/m² vs. 39 ± 12 ml/m², *p* = 0.069) compared to those with RVIP LGE (*n* = 15) (Table 4). Additionally, they trended toward higher hs-TnT (8.8 ± 1.7pg/ml vs. 6.5 ± 1.8pg/ml, *p* = 0.066) compared to the RVIP group. NT-proBNP and CK levels were similar (Table 4).

**Table 4:** Comparison of male cyclists with RVIP vs. non-RVIP LGE

-	Non-RVIP LGE cyclists (*n* = 4)	RVIP LGE cyclists (*n* = 15)	*P*-value	Cohen’s d
Clinical parameters				
Age, yrs	47 ± 9	41 ± 6	0.304	0.84
Weight, kg	84 ± 14	79 ± 8	0.519	0.57
Height, m	1.79 ± 0.02	1.83 ± 0.07	0.109	0.56
BMI, kg/m²	26.2 ± 4.2	23.7 ± 1.9	0.315	1.03
BSA, m²	2.03 ± 0.14	2.01 ± 0.12	0.789	0.18
Body Fat, %	17 ± 9	15.0 ± 5	0.74	0.28
Hematocrit, %	43 ± 3	43 ± 3	0.929	0.05
hs-TnT, pg/ml	8.8 ± 1.7	6.5 ± 1.8	0.066	1.28
NT-proBNP, pg/ml	36.8 ± 49.7	33.4 ± 54.4	0.912	0.06
CK, U/l	182 ± 50	158 ± 81	0.48	0.32
Exercise test				
Systolic BP at rest, mmHg	118 ± 10	122 ± 8	0.405	0.57
Diastolic BP at rest, mmHg	80 ± 8	81 ± 5	0.773	0.23
Peak systolic BP, mmHg	213 ± 15	205 ± 19	0.434	0.41
Peak diastolic BP, mmHg	83 ± 5	86 ± 9	0.334	0.41
Heart rate at rest, bpm	47 ± 12	50 ± 8	0.645	0.37
Peak heart rate, bpm	168 ± 25	183 ± 10	0.337	1.06
VO_2_max, ml/kg/min	57 ± 2	57 ± 11	0.863	0.05
Maximal power, W	429 ± 50	437 ± 51	0.786	0.16
Cardiac MRI parameters				
LVEF, %	57 ± 7	62 ± 6	0.349	0.68
LV mass index, g/m^2^	90 ± 5	74 ± 10	0.001	1.70
LVEDVi, ml/m²	133 ± 16	108 ± 12	0.043	1.91
LVESVi, ml/m²	56 ± 9	42 ± 9	0.040	1.63
LVSVi, ml/m²	77 ± 16	67 ± 9	0.303	0.97
LAVi, ml/m²	48 ± 7	39 ± 12	0.069	0.85
RVEF, %	47 ± 4	57 ± 5	0.011	1.85
RVEDVi, ml/m²	144 ± 36	120 ± 15	0.286	1.16
RVESVi, ml/m²	76 ± 26	52 ± 11	0.161	1.66
RVSVi, ml/m²	67 ± 12	68 ± 9	0.959	0.04
RAVi, ml/m²	59 ± 40	46 ± 12	0.56	0.66
LGE size, %LV	4.2 ± 5.5	1.4 ± 0.7	0.382	1.18
LGE mass index, g/m²	3.1 ± 4.1	0.8 ± 0.4	0.335	1.33
Native T1, ms	1240 ± 68	1220 ± 36	0.663	0.38
Native T2, ms	47 ± 1	48 ± 6	0.41	0.26
ECV, %	28 ± 1	26 ± 4	0.758	0.31
Cycling history				
Active years, n	23 ± 21	12 ± 6	0.382	1.05
Exercise volume, hours/week	11 ± 6	10 ± 5	0.81	0.16
Competitions n/year	17 ± 16	10 ± 6	0.442	0.80
Mean km/year	14200 ± 6800	7990 ± 2920	0.165	1.59
Competitiveness score	55 ± 17	57.8 ± 12.8	0.771	0.21

Numbers are mean ± SD for continuous and n (%) for categorical data. Cohen’s d indicates effect size (0.2 = small, 0.5 = medium, 0.8 = large, very large > 1.2) Notes: BMI, body mass index; BSA, body surface area; BP, blood pressure; CK, Creatine kinase; ECV, extracellular volume; HR, heart rate, hs-TnT, high-sensitive troponin T; LAVi, Left atrial end-systolic volume index; LV, left ventricular; LGE, late gadolinium enhancement; LVEF, left ventricular ejection fraction; LVEDVi, left ventricular end-diastolic volume index; LVESVi, left ventricular end-systolic volume index; LVSVi, left ventricular stroke volume index; RAVi, Right atrial end-systolic volume index; RVEF, right ventricular ejection fraction; RVEDVi, right ventricular end-diastolic volume index; RVESVi, right ventricular end-systolic volume index; RVSVi, right ventricular stroke volume index; NT-proBNP, N-terminal pro-brain natriuretic pepetide; VO_2max_, maximal oxygen uptake

The BSA, BMI, exercise test parameters, cycling history and competitiveness scores were not significantly different between the two groups (Table 4).

### Female vs. male cyclists

The mean age of female cyclists was similar to that of male cyclists (Table 5). The BSA and BMI were lower in females compared to males (BSA: 1.78 ± 0.17m^2^ vs. 1.99 ± 0.13m^2^, *p* = 0.009; BMI: 21.4 ± 2.1 kg/m^2^ vs. 23.9 kg/m^2^ ± 2.6, *p* = 0.013). However, body fat percentage did not differ significantly (17 ± 4% for females vs. 16 ± 5% for males, *p* = 0.522). Female cyclists had a significantly lower hematocrit than male cyclists (40 ± 2% vs. 44 ± 3%; *p* < 0.001). Hs-TnT, NT-proBNP and CK levels were similar (Table 5).

**Table 5:** Comparison of male and female cyclists

-	Female cyclists (*n* = 8)	Male cyclists (*n* = 58)	*P*-value
Clinical parameters			
Age, yrs	35 ± 13	43 ± 12	0.159
Weight, kg	65 ± 10	79 ± 9	0.007
Height, m	1.74 ± 0.07	1.81 ± 0.06	0.026
BMI, kg/m²	21.4 ± 2.1	23.9 ± 2.6	0.013
BSA, m²	1.78 ± 0.17	1.99 ± 0.13	0.009
Body Fat, %	17 ± 4	16 ± 5	0.522
Hematocrit, %	40 ± 2	44 ± 3	< 0.001
hs-TnT, pg/ml	4.6 ± 1.1	5.8 ± 2.2	0.088
NT-proBNP, pg/ml	40.6 ± 17.8	30.1 ± 32.6	0.191
CK, U/l	121 ± 85	164 ± 166	0.262
Exercise test			
Systolic BP at rest, mmHg	117 ± 9	124 ± 10	0.075
Diastolic BP at rest, mmHg	81 ± 3	80 ± 5	0.371
Peak systolic BP, mmHg	187 ± 24	211 ± 24	0.025
Peak diastolic BP, mmHg	87 ± 18	84 ± 7	0.68
Heart rate at rest, bpm	55 ± 7	52 ± 8	0.274
Peak heart rate, bpm	182 ± 15	180 ± 14	0.724
VO_2_max, ml/kg/min	52 ± 11	57 ± 10	0.348
Maximal power, W	317 ± 31	430 ± 53	< 0.001
Cardiac MRI parameters			
LVEF, %	64 ± 6	61 ± 5	0.278
LV mass index, g/m^2^	61 ± 7	79 ± 9	< 0.001
LVEDVi, ml/m²	100 ± 9	113 ± 12	0.004
LVESVi, ml/m²	36 ± 8	44 ± 8	0.039
LVSVi, ml/m²	64 ± 7	69 ± 8	0.0799
LAVi, ml/m²	37 ± 9	40 ± 11	0.462
RVEF, %	56 ± 6	55 ± 5	0.654
RVEDVi, ml/m²	107 ± 9	125 ± 17	< 0.001
RVESVi, ml/m²	47 ± 9	57 ± 13	0.027
RVSVi, ml/m²	60 ± 6	68 ± 8	0.007
RAVi, ml/m²	46 ± 13	52 ± 16	0.256
Native T1, ms	1170 ± 130	1220 ± 37	0.382
Native T2, ms	45 ± 4	47 ± 4	0.156
ECV, %	27 ± 3	26 ± 4	0.185
Cycling history			
Active years, n	5 ± 4	14 ± 10	< 0.001
Exercise volume, hours per week	10 ± 3	10 ± 4	0.844
Competitions per year, n	11 ± 9	14 ± 13	0.371
Mean km/year	8310 ± 3760	9350 ± 3490	0.479
Competitiveness score	65 ± 15	58 ± 14	0.213

Numbers are mean ± SD for continuous and n (%) for categorical data. Notes: BMI, body mass index; BSA, body surface area; BP, blood pressure; CK, Creatine kinase; ECV, extracellular volume; HR, heart rate, hs-TnT, high-sensitive troponin T; LAVi, Left atrial end-systolic volume index; LV, left ventricular; LGE, late gadolinium enhancement; LVEF, left ventricular ejection fraction; LVEDVi, left ventricular end-diastolic volume index; LVESVi, left ventricular end-systolic volume index; LVSVi, left ventricular stroke volume index; RVEF, right ventricular ejection fraction; RAVi, Right atrial end-systolic volume index; RVEDVi, right ventricular end-diastolic volume index; RVESVi, right ventricular end-systolic volume index; RVSVi, right ventricular stroke volume index; NT-proBNP, N-terminal pro-brain natriuretic pepetide; VO_2_max, maximal oxygen uptake

Peak systolic blood pressure during the exercise test was significantly lower in females than males (187 ± 24mmHg vs. 211 ± 24mmHg; *p* = 0.025). VO_2_max was not significantly different with 52 ± 11 ml/kg/min for females vs. 57 ± 10 ml/kg/min for males, *p* = 0.348. Maximal power output was lower in female cyclists compared to male cyclists (317 ± 31 W vs. 430 ± 53 W; *p* < 0.001).

Females had a lower LV mass index (61 ± 7 g/m² vs. 79 ± 9 g/m², *p* < 0.001), LVEDVi, (100 ± 9 ml/m² vs. 113 ± 12 ml/m², *p* = 0.004), LVESVi (36 ± 8 ml/m² vs. 44 ± 8 ml/m², *p* = 0.039), RVEDVi, (107 ± 9 ml/m² vs. 125 ± 17 ml/m², *p* < 0.001) and RVESVi (47 ± 9 ml/m² vs. 57 ± 13 ml/m², *p* = 0.027) compared to male cyclists. No differences in LVEF or RVEF were observed (Table 5). Mapping parameters did not differ between the group.

Female cyclists reported fewer active years in cycling (5 ± 4 years) compared to male cyclists (14 ± 10 years; *p* < 0.001), although weekly exercise volume, annual competition participation, mean kilometers per year, and competitiveness scores did not significantly differ between the two groups (Table 5).

## Discussion

In this study, we evaluated cardiac adaptations and LGE patterns in competitive male and female amateur cyclists using cardiac MRI. Consistent with the ‘athlete’s heart’, our cohort exhibited bi-ventricular enlargement and increased LV mass with preserved function compared to controls. Nearly one-third of male cyclists showed focal non-ischemic LGE, predominantly at the RVIP, while non-RVIP LGE was less frequent but associated with increased myocardial mass, reduced RVEF, and elevated hs-TnT levels. No significant associations were observed between focal LGE and peak-systolic blood pressure during exercise, fitness, cycling history, or competitiveness.

Cardiac MRI has become central to the assessment of structural and functional myocardial changes in athletes, yet cyclist-specific data remain limited [27, 28]. To our knowledge, this is the largest cardiac MRI study focusing exclusively on cyclists. Our cohort includes female cyclists, who were underrepresented in prior cyclist studies, more than doubles the number of male cyclists reported previously, and uses 3.0T MRI rather than the more commonly used 1.5T field strength in prior athlete studies, thereby providing reference data for future investigations into sport-specific cardiac adaptation in cycling [21, 27, 29].

The prevalence of LGE in our cohort is slightly higher than that reported in a recent smaller cardiac MRI study of professional cyclists [21], and falls within the range reported in runners, triathletes, and mixed-athlete populations [12]. RVIP LGE was the most common pattern in our cohort, consistent with previous studies that report its occurrence in up to 40% of endurance athletes [2, 30]. Of note, RVIP is also frequently observed in healthy non-athletes [29, 31]. Non-RVIP LGE was less prevalent, primarily located in the basal inferolateral LV wall, in line with previous studies in athletes [14]. Consistent with prior endurance studies, we found no evidence of diffuse myocardial fibrosis, as reflected by similar ECV in cyclists with and without focal LGE [29, 32].

In our cohort we observed that cyclists with non-ischemic non-RVIP LGE had higher LV volume and myocardial mass, reduced RVEF, a trend towards increased LA volume, and increased hs-TnT levels compared to cyclists without LGE or those with RVIP LGE only. The association of non-RVIP LGE with increased myocardial mass in our study aligns with findings of a triathlon study by Tahir et al. [6]. Interestingly, a recent study on soccer players and triathletes found that increased cardiac mass was associated with reduced global longitudinal strain, indicating that increased myocardial mass may have subclinical functional impact [33].

In contrast, cyclists with RVIP LGE only showed no significant increase in myocardial mass or changes in systolic function compared with cyclists without LGE. These findings reinforce the interpretation that isolated RVIP LGE represents a benign physiological adaptation rather than a pathological endpoint, a distinction that is further supported by the significant adverse remodeling observed exclusively in non-RVIP LGE subgroup. Notably, we did not observe the subtle functional decrements reported in younger elite athletes in the ELITE trial [2]. Given the older age of our cohort, this suggests that the minor functional variations may not progress to clinically meaningful remodeling or dysfunction over time, further reinforcing the likely benign nature of the RVIP LGE pattern.

The distinction between RVIP and non-RVIP LGE patterns is clinically relevant. The recent VENTOUX study of a large veteran mixed-athlete cohort reported that non-RVIP LGE, but not RVIP LGE, is associated with ventricular arrhythmias [1]. Our findings extended these observations by demonstrating that non-RVIP LGE in cyclists is associated not only with structural remodeling but also with elevated troponin levels, providing a potential pathophysiological link to the VENTOUX data on non-RVIP fibrosis and ventricular arrhythmia [1]. Together, these results support that non-RVIP LGE may represent a pathological substrate distinct from physiological athletic remodeling.

Elevated troponin levels in cyclists with LGE were observed in the absence of overt cardiovascular disease. While troponin elevation is typically linked to cardiomyocyte injury, emerging evidence suggests that stressed cardiomyocytes may release extracellular vesicles that could contribute to increased circulating troponin levels [34]. This suggests that cardiomyocyte stress, rather than acute injury or necrosis, may underlie the observed troponin elevations.

In contrast to a prior triathlete study [6], we observed no association between focal LGE, exercise-associated hypertension, or sports history, consistent with findings from mixed-athlete or marathon cohorts [29, 35]. Notably, we observed no association between LGE and cyclists’ competitiveness, a factor not previously explored in the literature. This underscores that the mechanisms leading to LGE in athletes are complex and cannot be solely attributed to training or competition intensity, highlighting the need for longitudinal studies to better define causal pathways.

Female cyclists in our cohort demonstrated high aerobic capacities comparable to their male counterparts, despite a shorter cycling history. Interestingly, none of the female cyclists exhibited LGE, which is within the range reported in previous endurance athlete studies [6, 12].

This study has several limitations. Cycling history was self-reported, which may introduce reporting and/or recall bias. Furthermore, despite pre-imaging exercise restriction, residual subclinical effects of recent training cannot be entirely excluded. Our collective consisted of competitive amateur cyclists, and the results may not be applicable to professional cyclists, who often accumulate substantially higher training volumes [36]. The number of female cyclists was limited; however, it reflects the current unequal gender distribution within cycling demographics in Germany , as exemplified by the German Cycling Federation , where females represent approximately 25% of members [37] . In addition, it is important to acknowledge that our modest sample size of female participants may also have limited our ability to detect cases of myocardial fibrosis. Additionally, the cross-sectional design and lack of ambulatory ECG monitoring preclude the detection of paroxysmal arrhythmias or the assessment of long-term prognostic significance. To reduce bias from varying exercise modalities, we focused exclusively on cyclists; however, we acknowledge that different cycling disciplines—ranging from prolonged endurance efforts to short, intense bursts—may lead to distinct cardiac adaptations. The prevalence of RV insertion point LGE in the control group was higher than reported in some prior cardiac MRI studies. Although controls were required to be healthy, engage in less than 3 h of exercise per week, and not participate in structured athletic training, detailed matching for lifetime exercise exposure or prior competitive sports participation was not performed. Accordingly, residual differences in historical training load cannot be excluded. In addition, self-selection bias inherent to volunteer-based recruitment may have influenced control group characteristics.

In conclusion, focal non-ischemic LGE is common among male competitive amateur cyclists, primarily involving the RVIP. Our findings indicate that RVIP and non-RVIP LGE represent distinct phenotypes with RVIP LGE appearing consistent with benign remodeling, whereas non-RVIP LGE is associated with adverse cardiac remodeling, reduced systolic function, and markers of subclinical myocardial injury. These findings suggest that non-RVIP fibrosis should not be considered as a benign variant of the athlete’s heart and may warrant closer clinical surveillance. Further longitudinal studies are needed to clarify underlying mechanisms and long-term clinical significance.

## Supplementary Information

Below is the link to the electronic supplementary material.

Supplementary Material 1

## Data Availability

The data underlying this article cannot be shared publicly due to concerns for the privacy of individuals that participated in the study. The data will be shared on reasonable request to the corresponding author.

## Abbreviations

BMI: body mass index
BSA: body surface area
CK: creatine kinase
ECV: extracellular volume
EF: ejection fraction
hs-TnT: high-sensitivity troponin T
LGE: late gadolinium enhancement
LV: left ventricle
LVEDVi: left ventricular end-diastolic volume index
MRI: magnetic resonance imaging
NT-proBNP: N-terminal pro–B-type natriuretic peptide
RVIP: right ventricular insertion point
RV: right ventricle
RVEDVi: right ventricular end-diastolic volume index
VO_2_max: maximal oxygen uptake

## References

[1] Javed W, Botis I, Goh ZM et al (2025) Ventricular Arrhythmia and Cardiac Fibrosis in Endurance Experienced Athletes (VENTOUX). Circ Cardiovasc Imaging 18:e018470. 10.1161/CIRCIMAGING.125.018470 40675176 10.1161/CIRCIMAGING.125.018470 PMC12356567

[2] Verwijs SM, Van Hattum JC, Daems JJN et al (2025) Cardiac ventricular hinge point LGE is highly prevalent and sex-dependent in ELITE athletes. Circ Cardiovasc Imaging e017765. 10.1161/CIRCIMAGING.124.017765 41060822 10.1161/CIRCIMAGING.124.017765

[3] Szabo L, Brunetti G, Cipriani A et al (2022) Certainties and Uncertainties of Cardiac Magnetic Resonance Imaging in Athletes. J Cardiovasc Dev Dis 9:361. 10.3390/jcdd9100361 36286312 10.3390/jcdd9100361 PMC9604894

[4] Haskell WL, Lee I-M, Pate RR et al (2007) Physical Activity and Public Health: Updated Recommendation for Adults from the American College of Sports Medicine and the American Heart Association. Med Sci Sports Exerc 39:1423–1434. 10.1249/mss.0b013e3180616b27 17762377 10.1249/mss.0b013e3180616b27

[5] Androulakis E, Swoboda PP (2018) The Role of Cardiovascular Magnetic Resonance in Sports Cardiology; Current Utility and Future Perspectives. Curr Treat Options Cardiovasc Med 20:86. 10.1007/s11936-018-0679-y 30167977 10.1007/s11936-018-0679-y PMC6132733

[6] Tahir E, Starekova J, Muellerleile K et al (2018) Myocardial Fibrosis in Competitive Triathletes Detected by Contrast-Enhanced CMR Correlates with Exercise-Induced Hypertension and Competition History. JACC Cardiovasc Imaging 11:1260–1270. 10.1016/j.jcmg.2017.09.016 29248656 10.1016/j.jcmg.2017.09.016

[7] Maron BJ, Doerer JJ, Haas TS et al (2009) Sudden Deaths in Young Competitive Athletes: Analysis of 1866 Deaths in the United States, 1980–2006. Circulation 119:1085–1092. 10.1161/CIRCULATIONAHA.108.804617 19221222 10.1161/CIRCULATIONAHA.108.804617

[8] Lund GK, Leptin S, Ragab H et al (2024) Prognostic Relevance of Ischemic Late Gadolinium Enhancement in Apparently Healthy Endurance Athletes: A Follow-up Study Over 5 years. Sports Med Open 10:13. 10.1186/s40798-024-00680-1 38282168 10.1186/s40798-024-00680-1 PMC10822825

[9] Messroghli DR, Moon JC, Ferreira VM et al (2017) Clinical recommendations for cardiovascular magnetic resonance mapping of T1, T2, T2* and extracellular volume: A consensus statement by the Society for Cardiovascular Magnetic Resonance (SCMR) endorsed by the European Association for Cardiovascular Imaging (EACVI). J Cardiovasc Magn Reson 19:75. 10.1186/s12968-017-0389-8 28992817 10.1186/s12968-017-0389-8 PMC5633041

[10] Kellman P, Hansen MS (2014) T1-mapping in the heart: accuracy and precision. J Cardiovasc Magn Reson 16:2. 10.1186/1532-429X-16-2 24387626 10.1186/1532-429X-16-2 PMC3927683

[11] Ferreira VM, Schulz-Menger J, Holmvang G et al (2018) Cardiovascular magnetic resonance in nonischemic myocardial inflammation. J Am Coll Cardiol 72:3158–3176. 10.1016/j.jacc.2018.09.072 30545455 10.1016/j.jacc.2018.09.072

[12] Allwood RP, Papadakis M, Androulakis E (2024) Myocardial fibrosis in young and veteran athletes: evidence from a systematic review of the current literature. J Clin Med 13:4536. 10.3390/jcm13154536 39124802 10.3390/jcm13154536 PMC11313657

[13] Terry KJ, Narducci D, Moran B et al (2024) Myocarditis in athletes: risk factors and relationship with strenuous exercise. Sports Med 54:607–621. 10.1007/s40279-023-01969-z 38079080 10.1007/s40279-023-01969-z

[14] Małek ŁA, Bucciarelli-Ducci C (2020) Myocardial fibrosis in athletes-current perspective. Clin Cardiol 43:882–888. 10.1002/clc.23360 32189357 10.1002/clc.23360 PMC7403702

[15] Androulakis E, Mouselimis D, Tsarouchas A et al (2021) The role of cardiovascular magnetic resonance imaging in the assessment of myocardial fibrosis in young and veteran athletes: insights from a meta-analysis. Front Cardiovasc Med 8:784474. 10.3389/fcvm.2021.784474 34993239 10.3389/fcvm.2021.784474 PMC8724053

[16] OʼToole ML, Douglas PS (1995) Applied physiology of triathlon. Sports Med 19:251–267. 10.2165/00007256-199519040-00003 7604198 10.2165/00007256-199519040-00003

[17] Kim YJ, Chun H, Kim C-H (2013) Exaggerated response of systolic blood pressure to cycle ergometer. Ann Rehabil Med 37:364. 10.5535/arm.2013.37.3.364 23869334 10.5535/arm.2013.37.3.364 PMC3713293

[18] Caselli S, Serdoz A, Mango F et al (2019) High blood pressure response to exercise predicts future development of hypertension in young athletes. Eur Heart J 40:62–68. 10.1093/eurheartj/ehy810 30590485 10.1093/eurheartj/ehy810

[19] Carlén A, Lindow T, Cauwenberghs N et al (2024) Exercise systolic blood pressure response during cycle ergometry is associated with future hypertension in normotensive individuals. Eur J Prev Cardiol 31:1072–1079. 10.1093/eurjpc/zwae012 38204381 10.1093/eurjpc/zwae012

[20] Ghekiere O, Herbots L, Peters B et al (2023) Exercise-induced myocardial T1 increase and right ventricular dysfunction in recreational cyclists: a CMR study. Eur J Appl Physiol 123:2107–2117. 10.1007/s00421-023-05259-4 37480391 10.1007/s00421-023-05259-4 PMC10492712

[21] Maceira A, Valenzuela PL, Santos-Lozano A et al (2023) Myocardial fibrosis and coronary calcifications caused by endurance exercise? Insights from former professional cyclists. Med Sci Sports Exerc 55:151–157. 10.1249/MSS.0000000000003043 36136597 10.1249/MSS.0000000000003043

[22] Schulz-Menger J, Bluemke DA, Bremerich J et al (2013) Standardized image interpretation and post processing in cardiovascular magnetic resonance: Society for Cardiovascular Magnetic Resonance (SCMR) Board of Trustees Task Force on Standardized Post Processing. J Cardiovasc Magn Reson 15:35. 10.1186/1532-429X-15-35 23634753 10.1186/1532-429X-15-35 PMC3695769

[23] Levine BD (2008) VO2 max: what do we know, and what do we still need to know? J Physiol 586:25–34. 10.1113/jphysiol.2007.147629 18006574 10.1113/jphysiol.2007.147629 PMC2375567

[24] Roger GE, Reilly T, Eston RG, Reilly T (2009) Human body composition. In: Eston RG, Reilly T (eds) Kinanthropometry and Exercise Physiology Laboratory Manual: Tests, Procedures and Data: Anthropometry, 3rd edn. Routledge, London, UK

[25] Parizkova J (1961) Total body fat and skinfold thickness in children. Metabolism 10:794–807 14483890

[26] Rothman KJ (1990) No adjustments are needed for multiple comparisons. Epidemiology 1:43–46 2081237

[27] Kawel-Boehm N, Hetzel SJ, Ambale-Venkatesh B et al (2025) Reference ranges (normal values) for cardiovascular magnetic resonance (CMR) in adults and children: 2025 update. J Cardiovasc Magn Reson 101853. 10.1016/j.jocmr.2025.101853 10.1186/s12968-020-00683-3 PMC7734766 33308262

[28] D’Ascenzi F, Anselmi F, Piu P et al (2019) Cardiac magnetic resonance normal reference values of biventricular size and function in male athlete’s heart. JACC Cardiovasc Imaging 12:1755–1765. 10.1016/j.jcmg.2018.09.021 30553678 10.1016/j.jcmg.2018.09.021

[29] Banks L, Altaha MA, Yan AT et al (2020) Left ventricular fibrosis in middle-age athletes and physically active adults. Med Sci Sports Exerc 52:2500–2507. 10.1249/MSS.0000000000002411 32472930 10.1249/MSS.0000000000002411

[30] Domenech-Ximenos B, Sanz-de la Garza M, Prat-González S et al (2020) Prevalence and pattern of cardiovascular magnetic resonance late gadolinium enhancement in highly trained endurance athletes. J Cardiovasc Magn Reson 22:62. 10.1186/s12968-020-00660-w 32878630 10.1186/s12968-020-00660-w PMC7469354

[31] Grigoratos C, Pantano A, Meschisi M et al (2020) Clinical importance of late gadolinium enhancement at right ventricular insertion points in otherwise normal hearts. Int J Cardiovasc Imaging 36:913–920. 10.1007/s10554-020-01783-y 32026265 10.1007/s10554-020-01783-y

[32] Małek ŁA, Barczuk-Falęcka M, Werys K et al (2019) Cardiovascular magnetic resonance with parametric mapping in long-term ultra-marathon runners. Eur J Radiol 117:89–94. 10.1016/j.ejrad.2019.06.001 31307657 10.1016/j.ejrad.2019.06.001

[33] Starekova J, Thottakara T, Lund GK et al (2020) Increased myocardial mass and attenuation of myocardial strain in professional male soccer players and competitive male triathletes. Int J Cardiovasc Imaging 36:2187–2197. 10.1007/s10554-020-01918-1 32564331 10.1007/s10554-020-01918-1 PMC7568698

[34] Hammarsten O, Wernbom M, Mills NL, Mueller C (2022) How is cardiac troponin released from cardiomyocytes? Eur Heart J Acute Cardiovasc Care 11:718–720. 10.1093/ehjacc/zuac091 35972428 10.1093/ehjacc/zuac091 PMC9522257

[35] Ragab H, Lund GK, Breitsprecher L et al (2023) Prevalence and pattern of focal and potential diffuse myocardial fibrosis in male and female marathon runners using contrast-enhanced cardiac magnetic resonance. Eur Radiol 33:4648–4656. 10.1007/s00330-023-09416-3 36683089 10.1007/s00330-023-09416-3 PMC10289973

[36] Lucia A, Hoyos J, Chicharro JL (2001) Physiology of professional road cycling. Sports Med 31:325–337. 10.2165/00007256-200131050-00004 11347684 10.2165/00007256-200131050-00004

[37] Deutscher Olympischer Sportbund (2023) Bestandserhebung 2023 (1st digital edition version November 2023 1, 2023). Frankfurt am Main: DOSB. Available from https://cdn.dosb.de/user_upload/www.dosb.de/uber_uns/Bestandserhebung/Bestandserhebung_2023.pdf

