# Supplementary material for: Cardiac remodeling and myocardial late gadolinium enhancement in competitive amateur cyclists: insights from cardiac MRI
Source: Int J Cardiovasc Imaging. 2026 Apr 13;42(8):1469–81. doi: 10.1007/s10554-026-03693-x (PMC13433387; doi:10.1007/s10554-026-03693-x)
Supplement: Supplementary file 1 [file 10554_2026_3693_MOESM1_ESM.docx]

**Supplementary Material**

**Table S1** Cyclists' intrinsic motivation to compete was assessed using a study-specific questionnaire comprising 10 statements rated on a 7-point Likert scale (1 = strongly disagree to 7 = strongly agree). The total score, ranging from 10 to 70, indicates the level of competitive drive and win-orientation, with higher scores reflecting greater competitive motivation. Statements 1, 3, and 7 (marked with an asterisk ∗) were reverse scored to ensure that a higher total score consistently represents greater competitive motivation.

|  | Strongly Strongly Disagree Agree | | | | | | |
| --- | --- | --- | --- | --- | --- | --- | --- |
| Taking part in a competition, participating is more important to me than winning. (_*_) | 1 | 2 | 3 | 4 | 5 | 6 | 7 |
|  | ☐ | ☐ | ☐ | ☐ | ☐ | ☐ | ☐ |
| If someone is working on the same thing as me, I try to be faster or better. | 1 | 2 | 3 | 4 | 5 | 6 | 7 |
|  | ☐ | ☐ | ☐ | ☐ | ☐ | ☐ | ☐ |
| I don't mind if others of the same age have achieved more than me. (_*_) | 1 | 2 | 3 | 4 | 5 | 6 | 7 |
|  | ☐ | ☐ | ☐ | ☐ | ☐ | ☐ | ☐ |
| To have clarity on my performance, it's important for me to compare myself with others. | 1 | 2 | 3 | 4 | 5 | 6 | 7 |
|  | ☐ | ☐ | ☐ | ☐ | ☐ | ☐ | ☐ |
| The desire to be better than others is a great motivation for me. | 1 | 2 | 3 | 4 | 5 | 6 | 7 |
|  | ☐ | ☐ | ☐ | ☐ | ☐ | ☐ | ☐ |
| I enjoy competing against others. | 1 | 2 | 3 | 4 | 5 | 6 | 7 |
|  | ☐ | ☐ | ☐ | ☐ | ☐ | ☐ | ☐ |
| It bothers me when others perform better. | 1 | 2 | 3 | 4 | 5 | 6 | 7 |
|  | ☐ | ☐ | ☐ | ☐ | ☐ | ☐ | ☐ |
| I rarely felt like I was in competition with others. (_*_) | 1 | 2 | 3 | 4 | 5 | 6 | 7 |
|  | ☐ | ☐ | ☐ | ☐ | ☐ | ☐ | ☐ |
| When I see others can do more than I can, it motivates me to work harder in the future. | 1 | 2 | 3 | 4 | 5 | 6 | 7 |
|  | ☐ | ☐ | ☐ | ☐ | ☐ | ☐ | ☐ |
| To truly feel successful, I have to be better than everyone I compare myself to. | 1 | 2 | 3 | 4 | 5 | 6 | 7 |
|  | ☐ | ☐ | ☐ | ☐ | ☐ | ☐ | ☐ |

**Table S2** Cardiac MRI Protocol

| **Parameter** | **CINE bSSFP** | **T2 Mapping** | **T1 Mapping^a^** | **LGE** |
| --- | --- | --- | --- | --- |
| Acquisition matrix | 168x150 (SA) 168x152 (LA) | 152x138 | 152x149 | 220x180 |
| Slice thickness (mm) | 8 | 8 | 10 | 8 |
| Repetition time (ms) | 2.93 | 1000 | 2.9 | 6.1 |
| Echo Time (ms) | 1.46 | 10.7 |  | 2.9 |
| Echo train length | 8 | 81 | 100 | 20 |
| Flip angle (°) | 45 | 90 | 20 | 25 |
| Slice orientation | SA, LA | SA | SA | SA, LA |
| Acquisition Type | 2D | 2D | 2D | 2D |
| Notes | Balanced SSFP (sBTEE) | Black-blood GRASE (mGraSE BB) | MOLLI (bTFE readout) 5(3)3 | PSIR (TFE readout) |
| Notes: BH, breath-hold, LA, long axis (2-, 3- and 4-chamber); SA, short axis; TFE, turbo field echo ^a^ pre and post contrast (0.2 mmol/kg gadoterate meglumine (Dotarem, Guerbet, Sulzbach, Germany; rate 2.5 ml/s) | | | | |

**Table S3** Ventricular remodeling in cyclists with papillary muscles and prominent trabeculations included in myocardial mass.

| **CMR Parameter** | **Female**  **cyclists**  **(n=8)** | **Male**  **cyclists**  **(n=58)** | **Non-RVIP LGE cyclists**  **(n=4)** | **RVIP LGE**  **cyclists**  **(n=15)** | **LGE – cyclists (n=39)** |
| --- | --- | --- | --- | --- | --- |
| LVEF, % | 66± 5 | 62 ± 5 | 58 ± 7 | 61 ± 5 | 63 ± 5 |
| LV mass index, g/m² | 67 ± 8 | 86 ± 10 | 97 ± 5 | 82 ± 10 | 87 ± 10 |
| LVEDVi, ml/m² | 95 ± 9 | 105 ± 11 | 123 ± 16 | 100 ± 12 | 105 ± 8 |
| LVESVi, ml/m² | 33 ± 7 | 40 ± 8 | 52 ± 9 | 39 ± 8 | 39 ± 6 |
| LVSVi, ml/m² | 62 ± 6 | 65 ± 8 | 71 ± 15 | 61 ± 8 | 66 ± 7 |
| Data are given as mean ± SD.  Notes: LV, left ventricular; LVEF, left ventricular ejection fraction; LVEDVi, left ventricular end-diastolic volume index; LVESVi, left ventricular end-systolic volume index; LVSVi, left ventricular stroke volume index. | | | | | |

**Table S4** Comparison of male cyclists with RVIP LGE and without LGE (LGE-).

|  | **LGE –**  **cyclists**  **(n=39)** | **RVIP LGE**  **cyclists**  **(n=15)** | **P-value** |
| --- | --- | --- | --- |
| **Clinical parameters** |  |  |  |
| Age, yrs | 43 ± 14 | 41 ± 6 | 0.572 |
| Weight, kg | 78 ± 10 | 79 ± 8 | 0.617 |
| Height, m | 1.81 ± 0.06 | 1.83 ± 0.07 | 0.364 |
| BMI, kg/m² | 23.7 ± 2.6 | 23.7 ± 1.9 | 0.895 |
| BSA, m² | 1.98 ± 0.13 | 2.01 ± 0.12 | 0.443 |
| Body Fat, % | 16 ± 5 | 15 ± 5 | 0.569 |
| Hematocrit, % | 44 ± 3 | 43 ± 3 | 0.585 |
| hs-TnT, pg/ml | 5.1 ± 2.0 | 6.5 ± 1.8 | **0.040** |
| NT-proBNP, pg/ml | 28.2 ± 18.1 | 33.4 ± 54.4 | 0.731 |
| CK, U/l | 165 ± 197 | 158 ± 81 | 0.851 |
| **Exercise test** |  |  |  |
| Systolic BP at rest, mmHg | 125 ± 10 | 122 ± 8 | 0.349 |
| Diastolic BP at rest, mmHg | 79 ± 4 | 81 ± 5 | 0.19 |
| Peak systolic BP, mmHg | 213 ± 26 | 205 ± 19 | 0.203 |
| Peak diastolic BP, mmHg | 83 ± 6 | 86 ± 9 | 0.238 |
| Heart rate at rest, bpm | 53 ± 8 | 50 ± 8 | 0.235 |
| Peak heart rate, bpm | 180 ± 14 | 183 ± 10 | 0.434 |
| VO_2max_, ml/kg per min | 57 ± 10 | 57 ± 11 | 0.956 |
| Maximal power, W | 427 ± 55 | 437 ± 51 | 0.547 |
| **CMR parameters** |  |  |  |
| LVEF, % | 61 ± 4 | 62 ± 6 | 0.924 |
| LV mass index, g/m^2^ | 79 ± 8 | 74 ± 10 | 0.082 |
| LVEDVi, ml/m² | 112 ± 9 | 108 ± 12 | 0.237 |
| LVESVi, ml/m² | 43 ± 6 | 42 ± 9 | 0.501 |
| LVSVi, ml/m² | 69 ± 7 | 67 ± 9 | 0.327 |
| LAVi, ml/ m² | 40 ± 11 | 39 ± 12 | 0.842 |
| RVEF, % | 55 ± 5 | 57 ± 5 | 0.267 |
| RVEDVi, ml/m² | 125 ± 13 | 120 ± 15 | 0.286 |
| RVESVi, ml/m² | 56 ± 10 | 52 ± 11 | 0.181 |
| RVSVi, ml/m² | 68 ± 7 | 68 ± 9 | 0.851 |
| RAVi, ml/ m² | 53 ±14 | 46 ± 12 | 0.059 |
| Native T1, ms | 1210 ± 32 | 1220 ± 36 | 0.237 |
| Native T2, ms | 47 ± 3 | 48 ± 6 | 0.356 |
| ECV, % | 25 ± 3 | 26 ± 4 | 0.786 |
| **Cycling history** |  |  |  |
| Active years, n | 14 ± 10 | 12 ± 6 | 0.6 |
| Exercise volume, hours/week | 10 ± 3 | 10 ± 5 | 0.554 |
| Competitions n/year | 15 ± 14 | 10 ± 6 | 0.064 |
| Mean km/ year | 9370 ± 2880 | 7990 ± 2920 | 0.133 |
| Competitiveness score | 58 ± 14 | 58 ± 13 | 0.992 |
| Numbers are mean ±SD for continuous and n (%) for categorical data.  Notes: BMI, body mass index; BSA, body surface area; BP, blood pressure; CK, Creatine kinase; ECV, extracellular volume; HR, heart rate, hs-TnT, high-sensitive troponin T; LAVi, Left atrial end-systolic volume index; LV, left ventricular; LGE, late gadolinium enhancement; LVEF, left ventricular ejection fraction; LVEDVi, left ventricular end-diastolic volume index; LVESVi, left ventricular end-systolic volume index; LVSVi, left ventricular stroke volume index; RAVi, Right atrial end-systolic volume index; RVEF, right ventricular ejection fraction; RVEDVi, right ventricular end-diastolic volume index; RVESVi, right ventricular end-systolic volume index; RVSVi, right ventricular stroke volume index; NT-proBNP, N-terminal pro-brain natriuretic pepetide; VO_2max_, maximal oxygen uptake. | | | |
